# Clinical features and prognosis in hepatectomy for colorectal cancer with centrally located liver metastasis

**DOI:** 10.1186/s12957-015-0497-6

**Published:** 2015-03-04

**Authors:** I-Ming Kuo, Song-Fong Huang, Jy-Ming Chiang, Chien-Yuh Yeh, Kun-Ming Chan, Jinn-Shiun Chen, Ming-Chin Yu

**Affiliations:** Division of General Surgery, Department of Surgery, Chang Gung Memorial Hospital, Linkou, Chang Gung University, 5, Fu-Shin Street, Kweishan, Taoyuan, 333 Taiwan; Division of Colorectal Surgery, Department of Surgery, Chang Gung Memorial Hospital, Linkou, Chang Gung University, 5, Fu-Shin Street, Kweishan, Taoyuan, 333 Taiwan

**Keywords:** Colorectal cancer, Liver metastasis, Hepatectomy, Centrally located, Prognosis

## Abstract

**Background:**

Hepatic metastasectomy for patients with primary colorectal cancer offers better long-term outcome, and chemotherapy can increase the rate of hepatic resectability for patients with initially inoperable disease. The pattern of liver metastasis and status of the primary tumor are rarely discussed in the analysis of long-term outcome. In this report, we evaluate the influence of the pattern of metastasis on clinical features and prognosis.

**Methods:**

One hundred and fifty-nine patients who underwent hepatic metastasectomy with curative intent for liver metastasis of colorectal cancer between October 1991 and December 2006 were enrolled. Patients were grouped according to whether liver metastasis was centrally or peripherally located, based on imaging and operative findings. Patient demographics, characteristics of the primary and metastatic tumors, and surgical outcomes were analyzed for long-term survival.

**Results:**

A greater proportion of patients with centrally located metastases were male, as compared with those with peripherally located metastases. Compared with patients with peripherally located metastases, patients with centrally located metastases were more likely to have multiple lesions (*P* = 0.016), involvement of multiple segments (*P* = 0.006), large metastases (*P* < 0.001), and bilobar distribution of metastases (*P* < 0.001). The estimated 5-year recurrence-free and overall survival rates were 22.4% and 34.2%, respectively. Univariate analysis revealed that centrally located metastasis, primary tumor in the transverse colon, metastasis in regional lymph nodes, initial extrahepatic metastasis, synchronous liver metastasis, multiple lesions, poorly differentiated tumor, and resection margin <10 mm were significant poor prognostic factors for recurrence-free survival and overall survival. Cox regression analysis showed that inadequate resection margin and centrally located liver metastasis were significant predictors of shorter overall survival.

**Conclusions:**

In colorectal cancer, centrally located liver metastasis represents a poor prognostic factor after hepatectomy, and is associated with early recurrence. Neoadjuvant chemotherapy may be used to downstage centrally located liver metastases to improve outcome.

## Background

Half of all patients with colorectal cancer (CRC) develop liver metastases in the course of this disease [1-3]. Patients with colorectal liver metastases (CRLM) may benefit from liver resection because it provides an opportunity for curative management by means of repeated or staging hepatectomy [4-9]. Surgical strategies for CRLM and combined treatment to increase hepatic resectability in order to improve long-term outcome were reported by Adam *et al*. [10-14]. Earlier analyses of prognosis reported that multiple hepatic metastatic lesions, node-positive primary tumor, poorly differentiated primary tumor, extrahepatic spread, larger tumor size, higher carcinoembryonic antigen (CEA) level, and positive resection margin were significant predictors for shorter survival [15]. High CEA level, venous invasion, and tumor budding predicted extrahepatic recurrence after partial hepatectomy [16]. Fong *et al*. incorporated seven independent, significant predictors into a statistical model with a formula based on a 1,001 consecutive cases [17]. For patients undergoing hepatectomy for CRLM, achieving a resection margin of >1 cm has become standard in current clinical practice [18-20]. Adjuvant or salvage treatment with oxaliplatin-based or irinotecan-based regimen has been shown to improve outcome [21]. Whether the location of hepatic metastatic lesions influenced prognosis was rarely discussed. Centrally located liver metastases may affect the results of hepatectomy because of the restrictions of anatomy, technical difficulties in surgical approach, and morbidity resulting from surgery. Little is known about the differences between CRLM with central versus peripheral location with regard to characteristics of the primary tumor, characteristics of metastatic tumor, and the clinical course after liver resection.

The aim of the study was to compare the clinicopathologic characteristics, timing of recurrence, and surgical outcome in patients with centrally or peripherally located colorectal liver metastasis. Medical records of consecutive patients with CRLM undergoing potentially curative liver resection at our institution were reviewed to assess the long-term outcome and independent significant prognostic factors.

## Methods

### Patient population

One hundred and fifty-nine patients who underwent liver resection with curative intent for resectable CRLM were enrolled in this retrospective study at Chang Gung Memorial Hospital Linkou Medical Center (Taoyuan, Taiwan) between October 1991 and December 2006, with a follow-up period ranging from 0.9 to 246.6 months (median: 38.5 months). Coexistent extrahepatic lesions or intrahepatic lesions after downstaging by neoadjuvant chemotherapy were evaluated to confirm resectablility. Patients were divided into two groups based on the location of hepatic metastases, either central or peripheral. Central metastases were defined as those located at the first or second generation of hepatic bifurcation (close to the hepatic hilum). Patients with concurrent central and peripheral lesions were grouped with patients who had only central metastases. The metachronous lesion was defined as a lesion arising 3 months or more after resection of the primary colorectal cancer [22,23]. With the approval of the institutional review board (IRB 98-1881B), the clinicopathological characteristics, surgical management, and long-term outcomes of patients were analyzed and compared between the two groups.

### Hepatic resection for CRLM

For assessment of the clinical status of the primary colorectal cancer and liver metastasis, all patients underwent full evaluation with appropriate studies including chest roentgenography, abdominal computed tomography (CT), and/or liver ultrasonography before surgery. Hepatic resection with curative intent was defined as complete resection of all hepatic metastatic lesions with preservation of a sufficient liver remnant. In order to achieve better resection margins and to avoid unnecessary damage to vital structures, intraoperative ultrasonography was performed for localization of the tumors and to visualize the spatial relationship to Glisson’s sheath. Liver resections were performed using either the clamp-crush technique or the Cavitron Ultrasonic Surgical Aspirator (CUSA; Valleylab, Inc., Boulder, CO, USA). Hilar inflow control was not routinely applied for the transection of liver parenchyma. Patients received postoperative follow-up and were monitored for tumor recurrence by means of physical examination, serum CEA levels, and abdominal ultrasonography once every 3 months thereafter. Advanced image studies, including CT scan and/or magnetic resonance imaging (MRI) of the chest, abdomen, and pelvis, were performed when cancer recurrence was suspected.

### Data collection and statistical analysis

Information on demographics, characteristics of the primary and metastatic tumor, surgical details, and hospital course was collected from medical records. Patients who died within 30 days of liver resection or during the same hospitalization were considered to have suffered surgical mortality. Recurrence after liver resection was defined as the presence of a new lesion detected by an imaging study or a new lesion characterized by histological examination from either biopsy or surgical resection.

Statistical analyses were conducted using the statistical software SPSS 20.0 (IBM Corp., Armonk, NY, USA). Outcome measures included recurrence-free survival (RFS) and overall survival (OS) after liver resection. The *χ*^2^ test was used to compare clinicopathologic features. Continuous data were presented as the mean ± standard error of the mean (SEM) and were analyzed by the *t* test. RFS and OS were estimated using the Kaplan-Meier method, and any significant difference between the subgroups noted by univariate analysis was compared using the log-rank test. Multivariate analysis was conducted with the Cox regression. A *P* value of < 0.05 was defined as statistically significant.

## Results

### The characters of primary tumor, liver metastasis, and interval of resection in patients with central or peripheral metastases

One hundred and fifty of 159 patients (94.3%) were followed for more than 6 months after liver resection. Twenty-four patients (15.1%) presented with central liver metastases, while 135 patients (84.9%) had peripheral metastases. Table 1 summarizes demographic data. There were no significant differences in clinical characteristics between the two groups except for gender; men comprised a larger proportion of patients in the group with central metastases (79.2% vs. 51.9%, *P* = 0.014). There was no significant difference between the two groups in primary tumor location, tumor staging, regional lymph node metastasis, or bowel obstruction or perforation, but 18 patients (11.3%) had concurrent extrahepatic metastases at the time of hepatectomy (central vs. peripheral group, 25.0% vs. 8.9%, *P* = 0.033).

**Table 1:** **Patient demographics and the characteristics of the primary tumor(s)**

	**Centrally located**	**Peripherally located**	***P*** **values**
	**(** ***n*** **= 24)**	**(** ***n*** **= 135)**	
Gender			
Male	19 (79.2%)	70 (51.9%)	0.014
Female	5 (20.8%)	65 (48.1%)	
Age (year)	58.5 ± 2.4 (29–75)	59.7 ± 1.0 (24–86)	0.62
Location of the primary tumor(s)			
Rectum	13 (54.2%)	56 (41.5%)	0.541
Sigmoid colon	6 (25.0%)	28 (20.7%)	
Descending colon	1 (4.2%)	10 (7.4%)	
Transverse colon	3 (12.5%)	15 (11.1%)	
Ascending colon and cecum	1 (4.2%)	22 (16.3%)	
Synchronous^a^	0 (0.0%)	4 (3.0%)	
Primary tumor staging			
T1 or T2	2 (8.3%)	6 (4.4%)	0.437
T3	7 (29.2%)	56 (41.5%)	
T4	15 (62.5%)	73 (54.1%)	
Regional lymph node metastasis			
N0	6 (25.0%)	26 (19.3%)	0.705
N1	8 (33.3%)	56 (41.5%)	
N2	10 (41.7%)	53 (39.3%)	
Primary extrahepatic metastasis	6 (25.0%)	12 (8.9%)	0.033
Associated risky presentation(s)^b^	5 (20.8%)	16 (11.9%)	0.321

^a^Synchronous: two or more primary colorectal cancers identified at the same time; ^b^Associated risky presentation(s) included obstruction, tumor rupture/perforation, or both.

There were additional significant differences between the two groups with regard to liver metastases. The maximum diameter of the largest metastasis was greater in the group with central lesions (5.9 ± 0.8 vs. 3.0 ± 2.4 cm). Significant poor prognostic factors were more prevalent in the group with central lesions. These factors included the involved segment, lobar distribution, and number of metastasis. Hepatic metastases occurred synchronously in 104 patients (65.4%), and there was no difference between the two groups with regard to synchronicity. In addition, serum CEA level prior to hepatectomy and the degree of differentiation of metastatic lesions were not statistically significant; however, the CEA level was somewhat greater in the group with central lesions (366 ± 205 vs. 109 ± 53 ng/ml, *P* = 0.095) (Table 2).

**Table 2:** **The characteristics of liver metastasis**

	**Centrally located**	**Peripherally located**	***P*** **values**
	**(** ***n*** **= 24)**	**(** ***n*** **= 135)**	
CEA level before hepatectomy (ng/ml)	366.2 ± 205.5 (0.88 to 4,280.0)	109.9 ± 53.2 (0.50 to 7,025.0)	0.095
Interval of hepatic metastasis^a^			
Synchronous	17 (70.8%)	87 (64.4%)	0.544
Metachronous	7 (29.2%)	48 (35.6%)	
Involved segment(s)			
Single	3 (12.5%)	56 (41.5%)	0.006
Multiple	21 (87.5%)	79 (58.5%)	
Lobar distribution			
Unilobar	6 (25.0%)	108 (80.0%)	0.000
Bilobar	18 (75.0%)	27 (20.0%)	
Maximal diameter of the largest metastasis (cm)	5.9 ± 0.8 (1.2 to 17.9)	3.0 ± 2.4 (0.3 to 10.8)	0.000
Number of metastasis			
Solitary	10 (41.7%)	91 (67.4%)	0.016
Multiple	14 (58.3%)	44 (32.6%)	
Differentiation of metastasis			
Well	5 (20.8%)	10 (7.4%)	0.113
Moderately	18 (75.0%)	120 (88.9%)	
Poorly	1 (4.2%)	5 (3.7%)	

^a^The metachronous type was defined as when the metastasis was noted for 3 months or more after resection of primary colorectal cancer.

### Management and surgical results

Fifteen patients (9.4%) received neoadjuvant chemotherapy with 5-fluorouracil-based regimens. Disease progression occurred in nearly three quarters of all patients in the present study. Anatomic resection with curative intent was performed in patients with multiple lesions or lesions that were difficult to resect, but there was no difference between the two groups (29.2 vs. 18.5%, *P* = 0.27). In the group with central lesions, 41.7% of patients had resection margin involved with tumor even though surgical resection was performed with attention to achieving a grossly negative margin (*P* = 0.003). There was no difference in the recurrence pattern between the two groups, but patients whose metastases were centrally located were more likely to have early recurrence, with an interval of less than 4 months (37.5% vs. 17.8%, *P* = 0.032). One patient with peripheral metastatic tumor died in the hospital because of leakage at the anastomotic site and resultant sepsis; however, the liver resection *per se* was without intraoperative or postoperative complication (Table 3).

**Table 3:** **Management, resection margin, and recurrence status after hepatectomy**

	**Centrally located**	**Peripherally located**	***P*** **values**
	**(** ***n*** **= 24)**	**(** ***n*** **= 135)**	
Neoadjuvant chemotherapy	4 (16.7%)	11 (8.1%)	0.247
Effective (stable, partial/complete response	0 (0.0%)	4 (36.4%)	0.159
Progression	4 (100%)	7 (63.6%)	
Anatomic resection^a^	7 (29.2%)	25 (18.5%)	0.269
Resection margin (mm)			
Involved	10 (41.7%)	20 (14.8%)	0.003
<10 mm	13 (54.2%)	85 (63.0%)	
≥10 mm	1 (4.2%)	30 (22.2%)	
Recurrence after hepatectomy	22 (91.7%)	100 (74.1%)	0.045
Confined in the liver, lung, or both	7 (31.8%)	31 (31.0%)	1.000
Recurrence in other distant organ(s)	15 (68.2%)	69 (69.0%)	
Early recurrence^b^	9 (37.5%)	24 (17.8%)	0.032
Repeated hepatectomy	1 (4.2%)	13 (9.6%)	0.696

^a^Anatomic resection includes left or right lobectomy, extended left/right lobectomy, or lateral segmetectomy; combination with other hepatectomy methods is excluded; ^b^Early recurrence: less than 4 months after the hepatectomy for the first liver metastasis.

### Recurrence-free and overall survival

**Figure 1:**
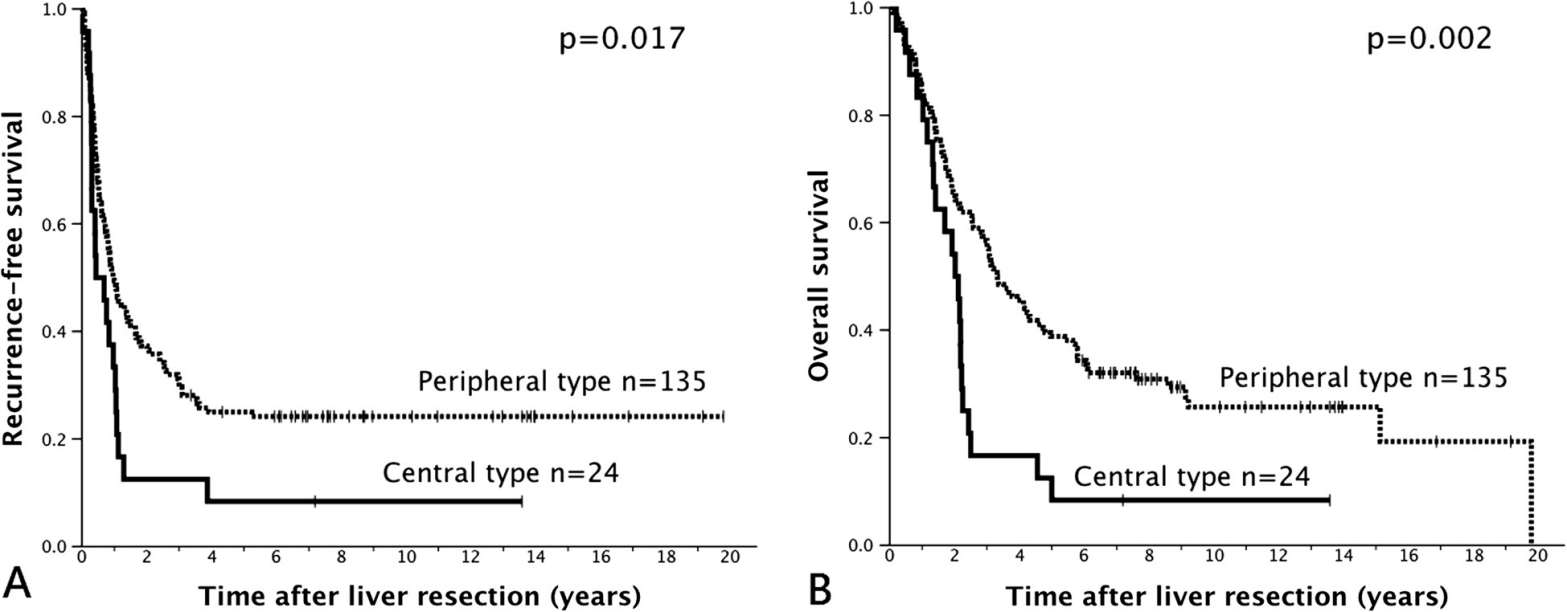
**Recurrence-free survival (A) and overall survival (B) of liver resection for central and peripheral liver metastases.**

For all patients in this study, the median time to recurrence was 10.4 months, and median survival time was 36.0 months. The estimated 5-year recurrence-free survival rate was 22.4%, and 5-year overall survival rate was 34.2%. Univariate analysis of prognostic factors for recurrence-free and overall survival revealed that the patients with central metastases had shorter recurrence-free (*P* = 0.017) and overall survival (*P* = 0.002) than did those with peripheral metastases (Figure 1). Primary tumor of the transverse colon, regional lymph node metastasis, primary extrahepatic metastasis, synchronous liver metastasis, multiple lesions and poorly differentiated metastatic lesions, and hepatectomy with margin of resection <10 mm were prognostic factors for shorter recurrence-free and overall survival in univariate analysis. In addition, advanced status of the primary tumor (T4), presentation with obstruction or perforation, involvement of multiple segments, and the presence of a metastatic lesion >5 cm in diameter were significant prognostic factors for shorter overall survival (Table 4).

**Table 4:** **Univariate analysis of prognostic factors on recurrence-free and overall survival**

	**Number of patient**	**5-year recurrence-free survival (%)**	***P*** **values**	**5-year overall survival (%)**	***P*** **values**
Overall	159	20.8		34.6	
				39.6	(from diagnosis of primary CRC)
Gender					
Male	89	19.5	0.699	31.5	0.449
Female	70	26.2		37.7	
Location of the primary tumor(s)					
Transverse colon	18	6.1	0.020	16.7	0.014
Other area (inclusive of synchronous)	141	24.4		36.4	
Primary tumor staging					
T1, T2, or T3	71	29.0	0.057	42.9	0.017
T4	88	17.3		27.3	
Regional lymph node metastasis					
Negative	32	41.7	0.002	48.4	0.004
Positive	127	17.7		30.7	
Primary extrahepatic metastasis					
Negative	141	25.1	0.000	37.9	0.000
Positive	18	0.0		5.6	
Associated risky presentation(s)					
Negative	138	23.5	0.424	38.0	0.047
Positive	21	15.1		9.5	
Interval of liver metastasis					
Metachronous	55	38.9	0.001	48.1	0.001
Synchronous	104	13.8		26.9	
Involved hepatic segment(s)					
Single	59	27.7	0.054	40.7	0.040
Multiple	100	19.3		30.3	
Metastatic tumor distribution					
Unilobar	114	24.1	0.193	37.2	0.200
Bilobar	45	18.2		26.7	
Metastatic tumor location					
Peripherally located	135	25.0	0.017	38.8	0.002
Centrally located	24	8.3		8.3	
Maximal diameter of the largest metastasis					
<5 cm	129	24.5	0.122	38.8	0.008
≥5 cm	30	13.9		13.8	
Number of liver metastasis					
Solitary	101	30.0	0.000	40.6	0.001
Multiple	58	9.0		22.8	
Differentiation of metastatic tumor					
Well differentiated	15	43.1	0.219 (well vs. moderately)	50.0	0.231 (well vs. moderately)
Moderately differentiated	138	21.1	0.002 (moderately vs. poorly)	34.1	0.000 (moderately vs. poorly)
Poorly differentiated	6	0.0	0.004 (well vs. poorly)	0.0	0.000 (well vs. poorly)
Mucinous adenocarcinoma					
Yes	13	23.1	0.813	38.5	0.922
No	146	22.4		33.8	
Neoadjuvant chemotherapy for liver metastasis					
Yes	15	20.0	0.677	35.7	0.477
No	144	22.8		20.0	
Anatomic resection for liver metastasis					
Yes	32	19.5	0.598	38.8	0.699
No	127	23.2		33.1	
Resection margin of metastatic tumor					
≥10 mm	31	40.2	0.013	58.1	0.002
<10 mm and involved	128	18.2		28.4	
Recurrence after liver resection					
Early recurrence (<4 months)	33			0.0	0.000
Late recurrence (≥4 months)	89			24.7	
Involved organ(s) while recurrent					
Confined in liver, lung or both	38	0.0	0.605	26.3	0.027
Other distant organ(s)	84	1.2		14.3	

**Figure 2:**
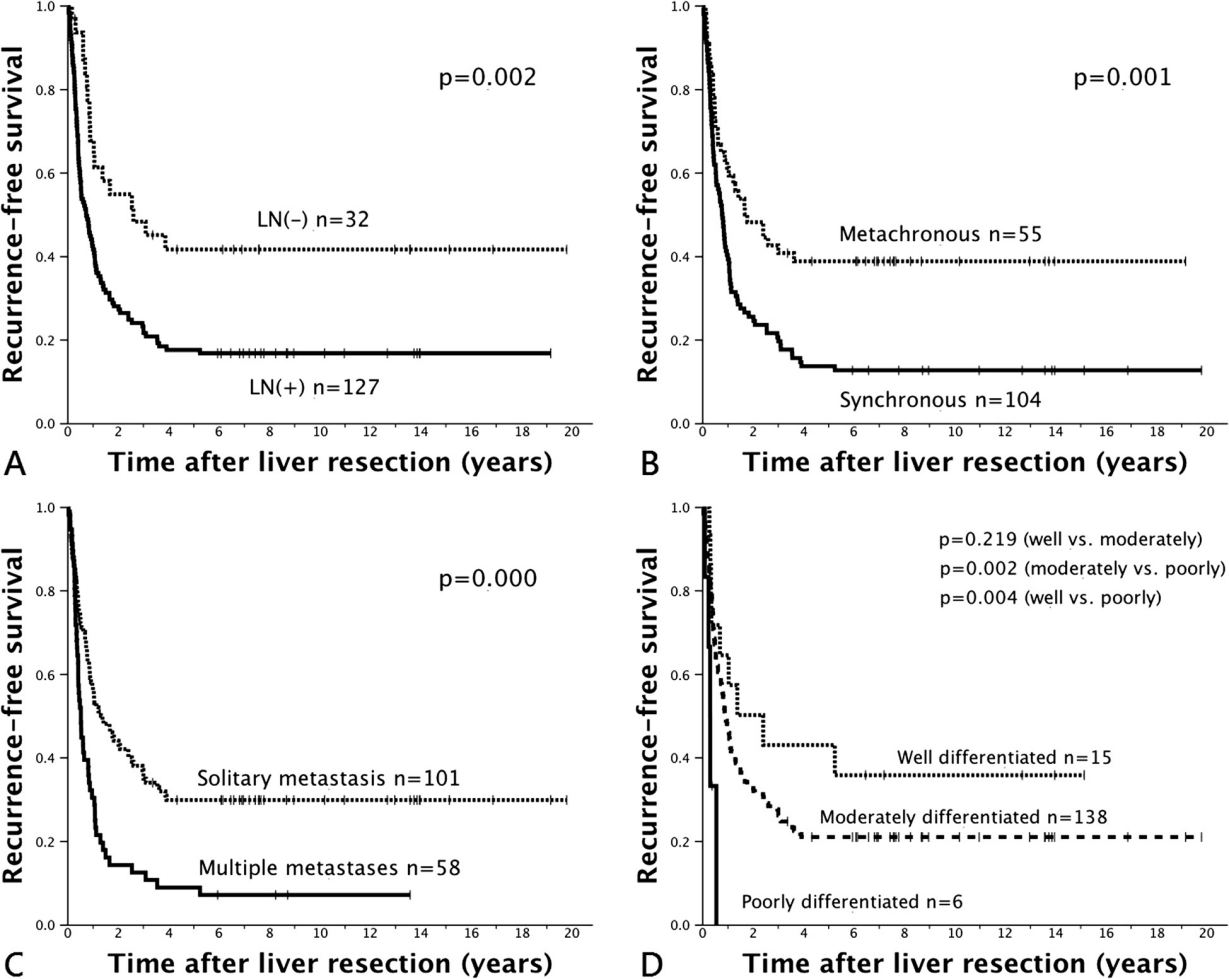
**Recurrence-free survival after liver resection in relation to independent significant prognostic factors. (A)** Regional lymph node(s) metastasis. **(B)** Synchronous versus metachronous lesions. **(C)** Number of hepatic metastatic lesions. **(D)** Grade of liver metastasis. Kaplan-Meier survival curves were plotted.

**Figure 3:**
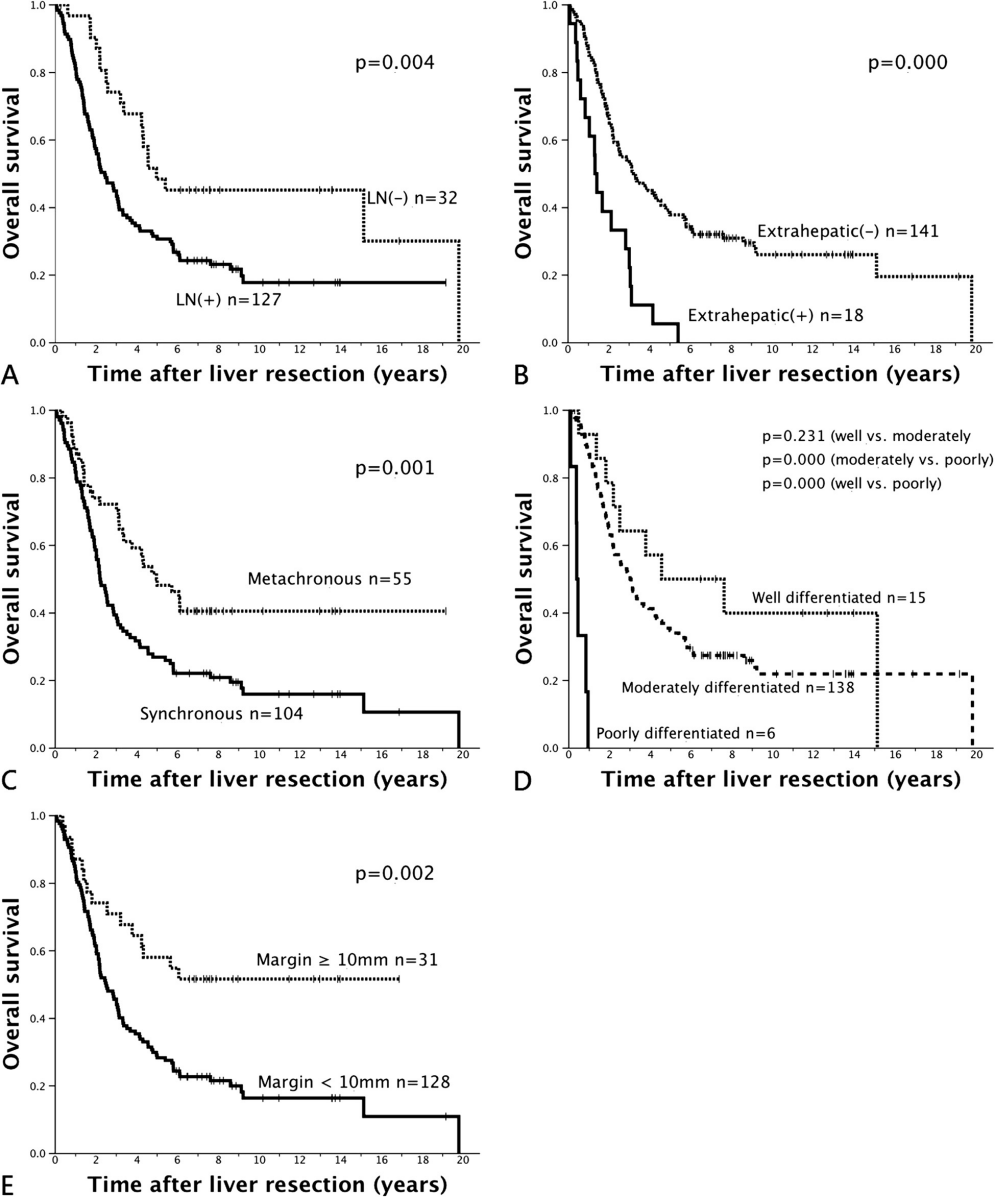
**Comparison of overall survival after liver resection based on the independent significant prognostic factors. (A)** Regional lymph node(s) metastasis. **(B)** Primary extrahepatic metastasis. **(C)** Synchronous versus metachronous metastases. **(D)** Grade of liver metastasis. **(E)** Resection margin of hepatectomy. Kaplan-Meier survival curves were plotted and examined by log-rank test between the two groups.

Cox regression analysis demonstrated that primary positive lymph node status, primary extrahepatic metastasis, synchronicity, central type hepatic metastasis, high grade of liver metastatic lesion, and resection margin of <1 cm all were independent significant prognostic factors that influenced overall survival. Each of these factors, except for primary extrahepatic metastasis and central location of lesions, was associated with worse prognosis in recurrence-free survival (Table 5). The estimated recurrence-free and overall survival curves based on each independent prognostic factor are shown in Figures 2 and 3.

**Table 5:** **Possible prognostic factors of recurrence-free survival (RFS) and of overall survival (OS) after liver resection (multivariate analysis)**

	**5-year RFS**		**5-year OS**	
	***P*** **values**	**Hazard ratio (CI 95%)**	***P*** **values**	**Hazard ratio (CI 95%)**
Factors of primary status				
Nodal involvement	0.018	1.847 (1.111 to 3.071)	0.010	1.975 (1.177 to 3.313)
Primary extrahepatic metastasis	NS	-	0.026	1.870 (1.079 to 3.240)
Factors of liver metastasis				
Synchronous presentation	0.024	1.627 (1.067 to 2.482)	0.007	1.775 (1.168 to 2.697)
Centrally located type	NS	-	0.036	1.705 (1.034 to 2.810)
Multiple lesions	0.018	1.601 (1.083 to 2.367)	NS	-
Poorly differentiation	0.029	2.899 (1.114 to 7.545)	0.000	9.284 (3.442 to 25.042)
Factors after liver resection				
Resection margin < 10 mm	NS	-	0.019	1.954 (1.118 to 3.414)

NS, not significant.

## Discussion

The location of liver metastasis from CRC might affect the surgical planning. In this series, most metastases were peripheral. Therefore, a single segmentectomy or subsegmentectomy, by Brisbane classification, was the most common surgical strategy. The outcome was relatively good even in the cases of multiple lesions because the hepatic functional reserve was adequate [24]; however, the precise influence of the location of liver metastasis is rarely evaluated and is not well described. Takasaki *et al*. introduced the Glissonean pedicle approach for anatomic resection in 1986. This approach can define the sections simply by clamping the extrahepatic pedicle [25]. In our study, central metastasis was defined as a metastatic lesion involving the area between the first and second generation of hepatic bifurcation. Sometimes, a lesion adjacent to the hepatic hilum, especially if it is large or irregularly shaped, may span both hepatic lobes or may involve more than one Couinaud segment.

In this study, there was no difference in the status of the primary tumor between the two groups, although gender distribution and presence of initial extrahepatic metastases differed between the groups. Patients with central metastases tended to have multiple lesions, involvement of multiple segments of the liver, bilobar distribution, and larger tumor size. These features were not related to synchronicity or tumor differentiation but definitely increased the difficulty of surgical treatment. We found that patients with central liver metastasis tended to have earlier recurrence than did patients with peripheral metastases and had markedly shorter survival (16.1 ± 1.9 vs. 43.0 ± 3.2 months, *P* < 0.001). When patients with CRLM underwent partial hepatectomy, the resection was performed with attention to functional anatomy of the liver. This approach may be associated with better surgical outcomes and prognosis, but there remains some controversy [22,23,26-29]. In general, there was no benefit from anatomic resection without adequate margins of resection in patients with CRLM.

Based on the recent reports, the 5-year recurrence-free survival ranges from 16% to 22%, and 5-year overall survival is 28% to 58% for patients with CRLM after liver resection (Table 6) [4,6,23,30-32]. In the present study, recurrence after hepatectomy occurred in 122 patients (76.7%) and resulted in further mortality. We found that significant risk factors for recurrence in patients with CRLM who underwent liver resection included centrally located metastasis, primary tumor located in the transverse colon, metastasis in regional lymph nodes, primary extrahepatic metastasis, synchronous metastasis, multiple and poorly differentiated metastatic lesions, and hepatectomy with resection margin <10 mm. These factors might differ from those described in other studies because of differences in patient populations and inclusion criteria [30,31,33-38].

**Table 6:** **Long-term outcomes after hepatectomy for CRLM**

**Author**	**Year**	**Number of resection**	**5-year RFS (%)**	**5-year OS (%)**
Ueno [30]	2000	85	21	28
Choti [4]	2002	133	19	58
Abdalla [6]	2004	358	-	58
Adam [23]	2004	138	22	33
Tsai [31]	2006	155	16.8	41.1
Are [32]	2007	1,019	-	37
Current series	2013	159	22.4	34.2

Adequacy of margins of resection has been shown to be an important and significant prognostic factor in hepatectomy; a clear margin >1 cm offered the best surgical outcome [18-20,37]; however, even a surgical margin of <1 cm should be considered in patients with CRLM if a 1-cm margin is impossible because of the size or location of the metastasis [22,26]. To date, no other single modality or treatment is superior to surgical resection; thus, it should be performed to improve the long-term survival [32]. In our multivariate analysis, patients with central metastases tended to have inadequate margins of resection, an independent prognostic factor, in comparison to those with peripheral lesions (41.7% vs. 14.8%, *P* = 0.003); however, both margins of resection and tumor location were independent significant prognostic factors.

There is no consensus about the definition of metachronous disease. Synchronous metastases were defined as metastatic lesions detected by preoperative examinations or during resection of the primary cancer, and metachronous metastases were defined as cancer arising at different times (from 3 months to 1 year) after diagnosis of the primary tumors [39]. With the advance of health screening and preoperative diagnostic tools, the synchronous lesion can be identified more accurately. In this study, metachronous metastases were defined with liver lesions at the interval of 3 months or more [22,23]. The patients with synchronous lesions tended to have higher CEA level, multiple lesions, and distant metastases if recurrence. The patients in the metachronous group experienced better 5-year recurrence-free and overall survival. These findings represented the character of dissemination and the tendency of leading to worse prognosis in the synchronous group of patients [31]. Higher anastomotic leakage rate after simultaneous resection of synchronous colorectal liver metastasis was disclosed in a recent study [40]. Excessive surgical stress resulted from longer operative time and more blood loss was possibly correlated with postoperative complications. Although one patient with peripheral and synchronous metastatic tumor died in the hospital because of sepsis from leakage at the anastomotic site, there was no definite intraoperative or postoperative complication from hepatic resection in our series.

It is interesting that when we selected the subgroup of patients with primary tumors of the transverse colon, this characteristic was shown by univariate analysis to be a significant risk factor in recurrence-free and overall survival. Hansen and Jess reported that cancers arising in the right-sided colon, and especially those arising in the transverse colon, had worse prognosis than did those arising in the left-sided colon [41]. Further basic investigation is needed to clarify the essential cellular and molecular mechanisms.

Patients with unresectable liver lesions benefited from hepatectomy after downstaging management [23]. Neoadjuvant chemotherapy can be applied to reduce the extent of metastasis with respect to tumor size, distribution, and numbers, thus improving the resectability [10-13]. Adam *et al*. proposed strategies to treat primary unresectable CRLM and methods to achieve downstaging as well as improvement in long-term outcome [14]. Systemic chemotherapy and/or targeted therapy are the standard protocol in both neoadjuvant therapy and sequential combination therapy after surgery [42,43]. However, few patients (*n* = 15) received neoadjuvant chemotherapy with 5-fluorouracil-based regimens, and most of them experienced disease progression in this study. The influence of neoadjuvant chemotherapy on prognosis could not be accurately analyzed in this study because of limited case numbers. The protocol was changed with update chemotherapy/targeted therapy regimens in our institute, and the role of central liver metastasis will be followed and analyzed with reducing bias statistical tools in the future. Because most cases of centrally located hepatic lesions were advanced, preoperative information can be provided more accurately by updating preoperative evaluations and newly developed techniques. Preoperative portal vein embolization to increase liver functional reserve is another option, although this technique is not yet widely applied in clinical practice [44]. Further studies involving neoadjuvant chemotherapy or portal embolization for downstaging and improving resectability will be evaluated.

In addition to patients benefiting from partial hepatic resection for hepatic metastases, some specific categories of patients with lung metastases from colorectal cancer can benefit from pulmonary resection [45-47]. However, Adam *et al*. report that hepatectomy for CRLM combined with lymphadenectomy does not benefit patients with involvement of distant lymph nodes, even when the disease is responsive to neoadjuvant chemotherapy [48]. That is to say, the impact of metastasis in CRC varies with the organs involved.

## Conclusions

In summary, central location of liver metastasis in CRC represented an independent significant prognostic factor for survival after hepatectomy. It affected the surgical outcome and was associated with early recurrence. Therefore, revision of treatment protocol such as newer chemotherapy regiment for downstaging for this higher risk group should be considered.
